# Production of biossurfactants using soybean meal andrice husk with *Bacillus amyloliquefaciens *mo-04b bysolid state fermentation (ssf)

**DOI:** 10.1186/1753-6561-8-S4-P223

**Published:** 2014-10-01

**Authors:** Juliana Massi, Doumit Camilios Neto, Maria Inês Rezende

**Affiliations:** 1 Universidade Estadual de Londrina, Londrina, PR, Brazil

## 

The biosurfactants are produced by various microorganisms and comprise a diversity of molecules characterized by low toxicity, high biodegradability and potential substitutes for synthetic surfactants that cause environmental impact. The southern region of Brazil has an agricultural based economy that produces large volumes of solid-waste per year. These solid-wastes must be properly treated and/or disposed. Alternatively, biotechnological strategies of use or re-use these solid-wastes have been applied aiming the production of metabolites with economic value. Given that, the production of biosurfactants by solid-state fermentation using agricultural-waste as substrates seems to be a promising option to decrease biosurfactant production costs. The goal of this study was to avaluate the capacity of production biosurfactants by *Bacillus amyloliquefaciens *MO-04B in solid state fermentation (FES), isolated from soil contaminated by oil collected near to the Getúlio Vargas Refinery-PR (PR-REPAR) and characterized as a producer of surfactin by submerged fermentation. The cultures were developed in 250 mL Erlenmeyer flasks containing soybean mean and rice husk 3:1g (w/w), dampened with a solution of salts (3 g/L KH_2_PO_4_, 7 g/L K_2_HPO_4_, 0.2 g/L MgSO_4_.7H_2_O; 1 g/L (NH_4_)_2_SO_4_, giving 80 % moisture; inoculated with 1 mL of cell suspension (4.10^8 ^CFU/mL) and incubated at 30 ± 2 °C for 30 h. The disruption was by adding 50 mL of distilled water content homogenised at 180 rpm for 30 min, and centrifuged at 10,000 rpm, 20 min., 4 ± 2 °C. In the supernatant were recovered by precipitation biosurfactants (6M HCl, pH 2) 4 ± 2 °C (overnigth) and extracted with dichloromethane. The sample was resuspended in water, neutralized (0.5 M NaOH, pH 7) and lyophilized. The performance of biosurfactants was 33 mg/GDS were solubilized in determining the emulsification index (IE_24_) equal to 62.5 % and the lowering of surface tension (ST) which was 39.6 mN/m. Thus *Bacillus amyloliquefaciens *MO-04b produced biosurfactants (lipopeptides/surfactin) by solid state fermentation substrates using soybean meal and rice husk as substrates. Futher studies will be developed to optimize the production of biosurfactants using factorial design and analysis by response surface methodology.

